# Phytochemical Analysis, Antioxidant and Analgesic Activities of *Incarvillea compacta* Maxim from the Tibetan Plateau

**DOI:** 10.3390/molecules24091692

**Published:** 2019-04-30

**Authors:** Jiajia Guo, Dan Zhang, Chao Yu, Ling Yao, Zhuo Chen, Yanduo Tao, Weiguo Cao

**Affiliations:** 1 College of Traditional Chinese Medicine, Chongqing Medical University, Chongqing 400016, China; gjj199509@163.com (J.G.); zhangdan01234567@sina.com (D.Z.); michealle_10@163.com (L.Y.); 15095835920@163.com (Z.C.); 2 College of Pharmacy, Chongqing Medical University, Chongqing 400016, China; yuchao@cqmu.edu.cn; 3 Northwest Institute of Plateau Biology, Chinese Academy of Sciences, Xining 810000, China,

**Keywords:** LC-MS, anti-oxidation, analgesia, formalin test, Tibetan plant

## Abstract

*Incarvillea compacta* Maxim is a traditional Tibetan plant widely used to treat rheumatic pain and bruises. We conducted qualitative analyses by liquid chromatography-mass spectrometry and quantitative analyses of the total phenols, flavonoids, and alkaloids content of different extracts of *I. compacta* Maxim. Antioxidant and analgesic activity were analyzed. The results showed that the methanol extract had the highest content of the various ingredients. A total of 25 constituents were identified, of which compounds **1**–**23** were found for the first time in this plant. The water extract had the highest capacity to clear free radicals in the antioxidant test. The water extract had dose-dependent analgesic effects in the first and second phase in a formalin test. The latency of pain from a hot-plate test was augmented by the water extract when the dose was greater than or equal to 30 g/kg. The water extract significantly decreased the amount of writhing in a dose-dependent manner compared with the control group in the acetic acid-induced writhing test. These results showed that *I. compacta* Maxim is a new antioxidant and analgesic agent, and this study provides information on its ingredients for further study.

## 1. Introduction

*Incarvillea compacta* Maxim (ICM) is a perennial herbaceous plant belonging to the *Incarvillea* genus and the family Bignoniaceae; and it the basic component of a Tibetan medicine named “Ouqu”. ICM often grows on alpine grasslands at altitudes of 2600–4100 m and is distributed in Tibet, Qinghai, Yunnan, Sichuan, and Gansu [1]. The roots, flowers, and seeds of ICM can be used as medicines to treat stomach pain, jaundice, poor digestion, ear empyema, irregular menstruation, and hypertension [2]. There are 16 species of the genus of *Incarvillea* in the world and 12 species in China, most of which are endemic to China and often used as traditional folk medicines. 

The literature on the plants belonging to the genus *Incarvillea* was summarized. Alkaloids, ceramides, iridoids, flavonoids, and terpenoids were found to be the primary components of this genus [3]. For instance, Su et al. identified two new alkaloids from *Incarvillea mairei*: isoincarvilline and incargranine A [4]. Ceramides and a new glucoceramide were found by Luo et al. in the roots of *Incarvillea arguta* [5]. A new iridoid, called incarvillic acid, was obtained from *Incarvillea delavayi* [6]. As for ICM, 23 compounds were identified in it by Zhao et al., including five phenylpropanoid glycosides, three flavonoids, three iridoid glycosides, five triterpenes, two steroids, and four other compounds. Another five phenylethanoid glycosides were discovered by Wu et al. [7,8,9]. However, these results are not enough to provide a full understanding of the composition of ICM. To strengthen the application of ICM in various fields, more chemical information should be explored. 

In terms of its modern pharmacological effects, only the effect of inducing apoptosis and hepatoprotective activity of ICM have been studied [9,10,11]. In current studies, plants belonging to the *Incarvillea* genus were primarily studied as analgesic drugs, and their alkaloids play a main role in antinociception [12]. Therefore, it is worth exploring whether ICM, belonging to the same genus, has similar biological activities. Pain is one of the primary problems that plague patients and has been listed as one of the five vital signs that cause physical and spiritual torture, and instill fear in patients, which has become a serious social problem greatly affecting the quality of life [13]. Given the limitations of clinical drugs, which generate adverse reactions and side effects [14], finding a resource-rich natural medicine that can relieve pain is essential. This is one of the reasons we wanted to explore the analgesic activity of ICM. Excessive free radical oxidative stress damages the human body at multiple levels, which can expedite the ageing process and cause various diseases, including inflammation and pain [15,16]. Previous studies have shown that antioxidants can produce analgesic effects through peripheral mechanisms [17]. Therefore, investigating the antioxidant activity of ICM is indispensable and will supply a reference for clinical medication. 

In summary, for this study, we measured the total phenols (TPs), total flavonoids (TFs), and total alkaloids (TAs) content of ICM and identified its chemical composition using liquid chromatography coupled with electrospray ionization quadrupole time-of-flight mass spectrometry (LC-ESI-QTOF-MS/MS). The antioxidant activities of the extracts produced by several reagents were tested using four assays. Three tests, including the formalin test, hot-plate test, and acetic acid-induced writhing, were conducted to examine the analgesic power of the water extract of ICM. The goal was to explore the chemical components and pharmacological activity of ICM, furnish evidence for clinical therapy and facilitate its development in the pharmaceutical industry.

## 2. Results

### 2.1. Total Phenols, Flavonoids and Alkaloids Content

ICM was extracted with water, methanol, and acetone (giving wICM, mICM, and aICM fractions, respectively). The TPs, TFs, and TAs contents of the ICM extracts in different solvents are shown in Table 1. As the results show, significant differences were found among the concentrations of TAs, TFs, and TPs in different extracts (*p* < 0.05). The highest TAs, TFs, and TPs contents were found in mICM, followed by wICM and aICM. TFs were the most abundant ingredient in this plant (49.07 mg RE (rutin equivalent)/g dry weight for mICM). The second highest content was the TPs (29.24 mg GAE (gallic acid equivalent)/g dry weight for mICM). The content of TAs was lower than of the other two compounds, with a content of 5.01 mg BE (berberine equivalent)/g dry weight in mICM. 

### 2.2. Chemical Composition of Incarvillea Compacta Maxim

Detailed fragmentation patterns were generated by LC-ESI-QTOF-MS/MS in both the positive and negative ion modes to identify the constituents. The primary components in mICM were phenolic acids, flavonoids, and alkaloids. A total of 25 compounds were discovered, and their retention times (Rt), error (ppm), and molecular ions are shown in Table 2.

#### 2.2.1. Phenolic Acids

The free phenolic profile of ICM was determined using LC-ESI-QTOF-MS/MS in negative-ion MS spectra due to its better sensitivity and more observable phenolic acid peaks. The preliminary identification of several phenolic acids in the ICM extract was accomplished by comparing their characteristic MS fragment ions with those in the literature. When subjected to MS/MS analysis, peak 1 showed the presence of syringic acid (strong peak at *m*/*z* 197.0456), which was further verified by the formation of two fragment ions. One fragment ion [M − H-44]^−^ at *m*/*z* 153 was formed by the loss of the CO_2_ group, and the other fragment ion group at *m*/*z* 182 was produced by the loss of a [M − H-15]^−^ CH_3_ group at *m*/*z* 182 [18]. Peak 2 was tentatively assigned as *p*-hydroxybenzoic acid; fragmentation from [M − H]^−^ ions at *m*/*z* 92.0266 showed a loss of CO_2_ [19]. Peak 3 (at *m*/*z* 191.0561) exhibited a molecular ion peak that corresponded to quinic acid, which was verified using two fragment ions [M − H-H_2_O]^−^ and [M − H-CO-2H_2_O]^−^ at *m*/*z* 173 and 127, respectively [20]. Peak 23, with an [M − H]^−^ ion at *m*/*z* 153, which produced a fragmentation at *m*/*z* 109 due to the loss of CO_2_, was clearly identified as protocatechuic acid by comparison with the reference. Combined with this result, we ascertained that peak 4 was protocatechuic acid hexoside by examining the MS^2^ fragment at *m*/*z* 153.0568, which explains the existence of hexoside [21]. The tentative mass spectrum for caffeic acid showed the deprotonated molecule [M − H]^−^ ion at *m*/*z* 179.03 at 6.59 min. The major fragment ions were *m*/*z* 161.0 and 135.0, corresponding to the losses of water and carbon dioxide, respectively, from the precursor ion. The pseudo-molecular ions of coumaric acid (*m*/*z* 163.04) produced the major fragment ions at *m*/*z* 119.0, corresponding to the loss of carbon dioxide from the precursor ion [33].

#### 2.2.2. Flavonoids

Peak 5 was identified as rutin ([M − H]^−^ *m*/*z* 609.1461), for which the ion at *m*/*z* 301 is typical and has been reported previously [36]. Peaks 6 and 8 shared a similar fragment ion peak (*m*/*z* 285.03), and the MS^2^ fragment ion peaks were at *m*/*z* 303 and 308, which correspond to the loss of rutinoside and glucoside, respectively. The only difference was that they were attached to a different glycoside between the two compounds. Based on the comparison of the MS data and retention times with those reported [23], peaks 6 and 8 were identified as kaempferol-3-*O*-rutinoside (nicotiflorin) and kaempferol-3-*O*-glucoside(astragalin). Fragment ion peaks at *m*/*z* 301.03 were detected using the MS procedure, which suggested that it was likely quercetin due to previous research [29]. Peaks 7, 10, and 12 were confirmed as quercetin-3-*O*-glucoside, quercetin-3-glucosid-7-*O*-rhamnoside, and quercetin-3-*O*-robinobioside, respectively, which proved that the lost fragment ion is 162 (glucoside), 146 (rhamnoside), and 308 (robinobioside), respectively. Peak 25 (tR = 39.26 min) exhibited an [M − H]^−^ ion at *m*/*z* 269.0456, which only had an error value of −3.4 ppm, and its fragmentation showed fragment ions at *m*/*z* 151.0046 and 117.0351, consistent with the fragmentation of apigenin that had been previously reported [35]. A debris ion at *m*/*z* 269 was examined in peak 9, and the [M − H]^−^ was 431.0984, indicating a loss of glucoside. Therefore, peak 9 was considered to be apigenin-7-O-glucoside (apigetrin) [25]. For peak 13 (tR = 19.34 min), the molecular ion [M + H]^+^ was seen at *m*/*z* 317.9656 in the positive mode. The primary MS^2^ fragmentation ions at *m*/*z* 317.0645 and 285.0360 were found, which were consistent with those previously reported in the literature [29]. In the positive mode, peak 14 displayed a molecular ion [M + H]^+^ at *m*/*z* 303.0497, and its daughters were *m*/*z* 285.0411, and 153.0188. Therefore, it was considered to be quercetin after comparison with other studies [29]. The presence of quercetin 3-glucoside in peak 15 was confirmed by the MS^2^ fragment ion at *m*/*z* 303.0485 that verified the appearance of quercetin, and the molecular ion [M + H]^+^ at *m*/*z* 465.1028 verified the loss of the glucoside. 

#### 2.2.3. Alkaloids and Others

**Figure 1:**
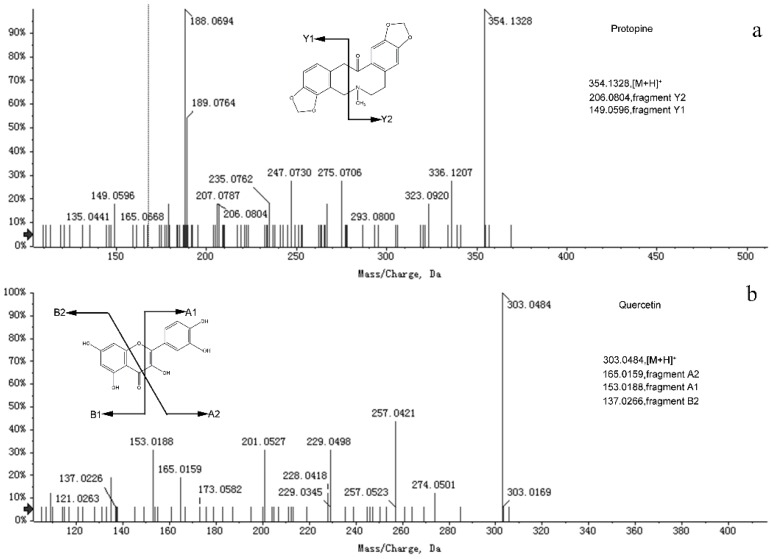
MS/MS spectra in positive mode of protopine (**a**) and quercetin (**b**).

The [M + H]^+^ ion at *m*/*z* 354 eluting at 11.13 min was observed to experience a Retro-Diels-Alder (RDA) fragmentation reaction; the fragment was observed at *m*/*z* 206.0804 and 149.0596. The neutral loss of H_2_O (*m*/*z* 206→188) was also detected; therefore, compound 11 was identified as protopine [27]. The fragmentation behaviors of protopine and quercetin are shown in Figure 1. At 1.69 min, the [M + H]^+^ ion at *m*/*z* 184 was detected. Its daughter ions at *m*/*z* 166 [Incarvilline-OH]^+^ were found in positive mode, which was the same as reported previously [30]. Therefore, peak 19 was identified as incarvilline. By comparison with the relevant literature, peak 20 was considered to be plantagonine. Peak 20 exhibited fragment ion [M + H]^+^ with *m*/*z* 178.0860. The ion with *m*/*z* 160.0752 lost the CO group, leading to the formation of an ion with *m*/*z* 132. By dissociating a methyl radical, this ion formed an ion with *m*/*z* 117.0568 [31]. Peak 21 showed an ion [M − H]^−^ at *m*/*z* 359.1348, whose daughter ions in negative mode were in accordance with preceding reports, which had been identified as 8-epideoxyloganic acid [32]. For peak 16 (Rt = 2.37), the molecular ion [M − H]^−^ was at *m*/*z* 133.01425 in negative mode. The primary MS^2^ fragmentation ions at *m*/*z* 115.0038, 71.0155, and 51.0191 of this compound were identified, which were consistent with those in the previous literature, and this compound was determined to be malic acid [23]. The [M − H]^−^ ion at *m*/*z* 191, which showed MS^2^ fragment ions at *m*/*z* 87 and 59, was identified as citric acid by comparison with a preceding paper [22]. Peak 18 displayed different fragment ions at *m*/*z* 149.0281, 133.0274, 105.0337, and 89.0396, which was consistent with previous articles. Hence, it was inferred to be esculetin [19]. 

### 2.3. Antioxidant Activity Analysis

#### 2.3.1. Scavenging Effect on DPPH Radicals

**Figure 2:**
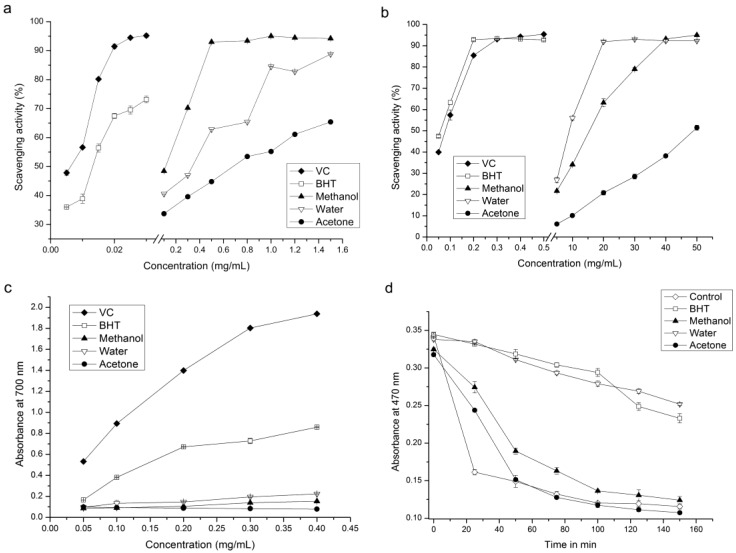
Antioxidant activity assays of ICM extract. (**a**) DPPH radical scavenging activity, (**b**) ABTS radical scavenging activity, (**c**) reducing power, (**d**) β-carotene/linoleic acid cooxidation activity.

The free radical scavenging activities of different extracts were measured using the DPPH (1,1-diphenyl-2-picrylhydrazyl) assay, and the results are shown in Figure 2a. The results showed that the antioxidant capacity of butylated hydroxytoluene (BHT) and ascorbic acid (VC) was similar, with IC_50_ values of 0.014 mg/mL and 0.013 mg/mL, respectively (*p* > 0.05). The different extracts were all effective at scavenging DPPH free radicals. The scavenging activities of these extacts increased with their increasing concentration. The scavenging rates of the mICM, wICM, and aICM were 48.45% to 94.21%, 40.56% to 88.80%, and 32.72% to 65.40%, respectively, when the extract concentration ranged 0.1 to 1.5 mg/mL. Compared with wICM and aICM, mICM had the highest ability to scavenge DPPH radicals based on the IC_50_ values of wICM, mICM and aICM being 0.532 mg/mL, 0.296 mg/mL, and 0.697 mg/mL, respectively, since a higher IC_50_ value suggests a lower antioxidant capacity.

#### 2.3.2. Scavenging Effect on ABTS Radicals

As shown in Figure 2b all the extracts demonstrated ABTS (2,2′-azino-bis(3-ethylbenzo-thiazoline-6-sulfonic acid)) free radical scavenging activity in a concentration-dependent manner. In the positive control group, the clearance of VC was stronger than that of BHT, with IC_50_ values of 0.122 mg/mL and 0.107 mg/mL, respectively (*p* < 0.05). Unlike the DPPH assay, wICM was the most effective at eliminating the ABTS free radical and had an IC_50_ value of 10.30 mg/mL, which was lower than that of mICM (19.491 mg/mL). The aICM was still the weakest scavenger because its scavenging rate only reached 51.45% at the highest concentration.

#### 2.3.3. Reducing Power Test

Reducing power is one of the mechanisms of antioxidant action, which may serve as a significant indicator of potential antioxidant activity [37]. The evaluation criterion of this method involves determining how much yellow Fe^3+^/ferricyanide can be reduced to green Fe^2+^ by a sample. The higher the content of Fe^2+^, the higher the absorbance, and the stronger the reduction ability [38]. The reducing powers of different samples are shown in Figure 2c. Compared with BHT, VC still has stronger a reducing ability. Although there was a disparity in reducing power among the three extracts, the difference was not obvious. Within the concentration range of 0.05 to 0.4 mg/mL, the absorbance values of the wICM, mICM, and aICM after reduction reaction were 0.096–0.223, 0.086–0.153, and 0.099–0.078, respectively. All the extracts were good electron donors and could reduce the Fe^3+^/ferricyanide complex to the Fe^2+^ form, which indicates antioxidant activity.

#### 2.3.4. β-Carotene Bleaching Test

The lipid antioxidant activity of extracts from ICM was evaluated using a β-carotene-linoleic acid bleaching assay. The β-carotene-linoleic acid bleaching assay is a hydrogen atom transfer reaction-based assay used to measure the ability of a compound or mixture to inhibit the oxidation of β-carotene [39]. As illustrated in Figure 2d, the absorbance in the control without antioxidants decreased significantly in 30 min. However, the absorbance values at 470 nm declined slowly with reaction time in the ICM extracts and BHT solution, and the solution consisting of water had a suppression ability similar to that of the positive control, which indicates that the water solution has a good antioxidant capacity. The acetone solution had almost no inhibitory ability and its curve almost coincided with that of the control group. 

### 2.4. Analgesic Test

#### 2.4.1. Formalin Test

**Figure 3:**
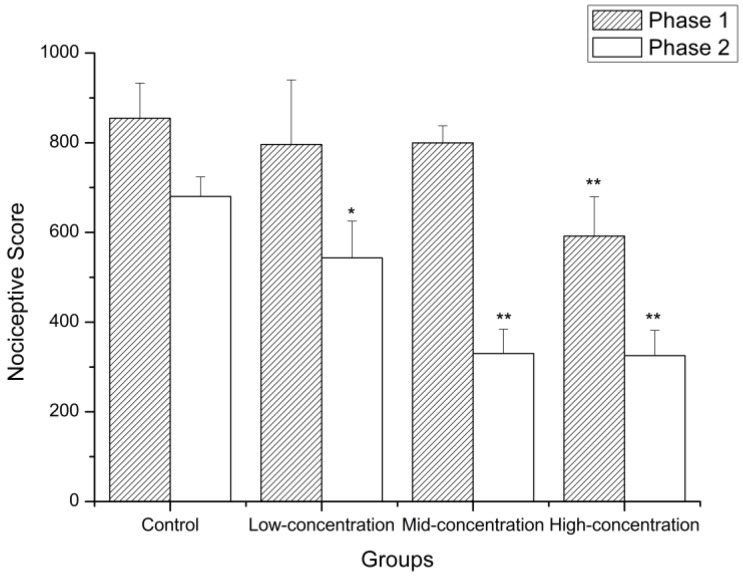
Analgesic effects of wICM in formalin test. Data are expressed as the means ± SD. * shows *p* < 0.05 compared with the control group; ** shows *p* < 0.01 compared with the control group.

The mouse formalin-induced pain model is an internationally recognized animal model for screening weak analgesic drugs. The pain response is divided into two phases, with each representing a different type of pain. The first phase is direct pain in the nerve endings and the second phase is caused by the production of peripheral stimuli and depends on the inflammatory reaction [40]. During the first phase, the animals treated with water had a nociceptive score of 854.67, and wICM at different concentrations (15, 30, and 60 g/kg) reduced the score to 796.00, 799.67, and 592.00, respectively (Figure 3). Previously, incarvillateine, found in plants of the *Incarvillea* genus, was considered to have strong analgesic activity at a concentration of 10–20 mg/kg [41]. Because the dosage in this study was calculated according to the amount of crude drug, it is reasonable that the concentration of ICM was much higher than that of incarvillateine, which was used as a standard. Only the high-dose group was significantly different from the control group (*p* < 0.01). During the second phase, the nociceptive score was also reduced by the addition of wICM in relation to the control group, and prominent differences were observed between all the treatment groups and the control group (*p* < 0.05). The score of the control and treatment groups are 680.33, 543.33 (low-concentration), 330.00 (mid-concentration), and 325.33 (high-concentration). All the results demonstrated that wICM significantly reduced the pain response in the second phase but had no significant effect in the first phase when treated with a lower concentration of wICM, indicating that wICM has more peripheral analgesic activity. 

#### 2.4.2. Hot-Plate Test

**Figure 4:**
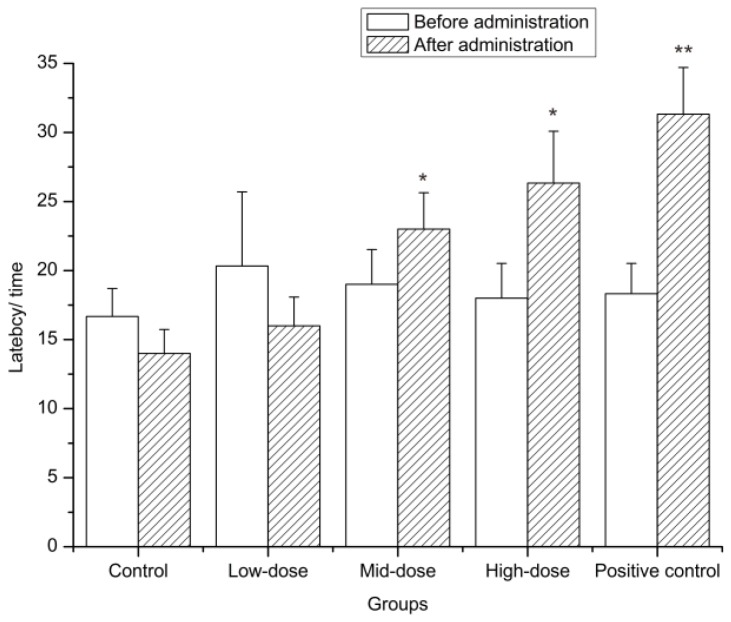
Antinociceptive effects of wICM in hot-plate test. Data are expressed as the means ± SD. * shows *p* < 0.05 compared with the control group; ** shows *p* < 0.01 compared with the control group.

To determine analgesic activity, the hot-plate test is used to measure the reaction time of the perception of pain. The heat stimulation sensitizes the peripheral nerve endings, and the impulses generated propagate to the brain via the spinal cord and result in a series of behaviours to avoid pain, which explains that the hot-plate test is suitable for evaluating analgesic effects related to nerves. Therefore, this test was conducted to examine the possible anti-nociceptive action of wICM. We found no difference in latency among these groups before the mice received treatment (*p* > 0.05) and the average latency was about 18.47 seconds. Compared to that of the control group, the latent periods of the positive control were significantly different from each other (*p* < 0.01) after administration. The wICM of the middle and high concentrations significantly extended the duration of pain (*p* < 0.05), which indicated that a higher concentration of wICM can protect mice from pain (Figure 4).

#### 2.4.3. Acetic Acid Writhing Test

**Figure 5:**
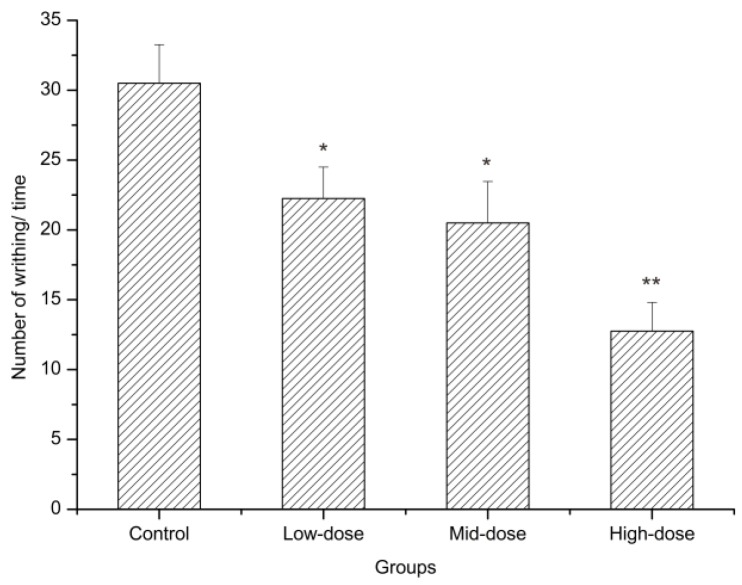
The effects of wICM on mice in acetic acid writhing test. Data are expressed as the means ± SD. * shows *p* < 0.05 compared with the control group; ** shows *p* < 0.01 compared with the control group.

The acetic acid writhing method is used to investigate peripheral analgesic activity. The effect of wICM on the amount of writhing in the mice is shown in Figure 5, Table 3. In this experiment, intraperitoneal (i.p.) injection of acetic acid produced a significant injury response to in distilled-water-treated mice. Administration of wICM 60 minutes before acetic acid injection led to a dose-dependent antinociceptive effect. All doses of the wICM noticeably lessened the number of writhing episodes when compared with the control group (*p* < 0.05). The analgesic rates of the low, middle and high doses of wICM were 27.05%, 32.79%, and 58.20%, respectively. Similar pharmacological effects were observed between the low and middle group (*p* > 0.05) and the high-dose was the most effective at reducing pain.

## 3. Discussion

### 3.1. Total Phenols, Flavonoids, and Alkaloids Content

Phenolic compounds, including flavonoids, widely exist in the plant kingdom and are important plant metabolites [42]. Alkaloids are the most studied compounds in the *Incarvillea* genus. Hence, these kinds of compounds were selected for determination. The extraction of chemical constituents from plants by solvents is based on the principle of similarity and intermiscibility, that is, chemical constituents dissolve in solvents with similar polarity. Therefore, the contents of the same kinds of components in different polar solvents will inevitably change. As shown by our results, the contents of different classes of components in different extracting solvents were different. The same conclusion, that the extraction amount of the same kind of components is significantly different in different polar solvents, was reached in a study on *Pistacia vera* var. Sarakhs [43]. Among the plants of the *Incarvillea* genus, the current research focuses on the investigation of alkaloids [44,45]. However, phenolic compounds and flavonoids are also important parts of the chemical constituents of ICM. Both flavonoids and phenols are closely related to many diseases [46]. Therefore, future research on ICM can develop in this direction.

### 3.2. Component Analysis

From the above results, the phenolic acids identified in this study can be classified into two categories: hydroxybenzoic acid and hydroxycinnamic acid [47]. The hydroxybenzoic acid and coumaric acid identified in this study have previously been found only in *Incarvillea delavayi*. Some studies reported that the same component in ICM, named protocatechuic acid, exists in the ethyl acetate soluble part of *I. delavayi* [48,49]. Although caffeic acid has not been found in other plants of the same genus, its esters have been identified in *Incarvillea mairei* [50]. It is inferred from the above that ICM and *I. delavayi* may be more similar in terms of components than others of the same genus. Research has reported that hydroxycinnamic acid amides are the main phenolic constituents in the reproductive organs of flowering plants [51]. ICM is a plant that can blossom and be used medicinally as a whole herb. However, no such compounds were identified in this study. The reason for this finding may be that amides belong to weak alkaline compounds with poor solubility in methanol. Hydroxycinnamic acid and its derivatives have been reported to show good antioxidant activity [52]. Oral administration of 4-hydroxybenzoic acid at low doses can also effectively improve the stress resistance of animals, and has anti-inflammatory and analgesic activities in traditional non-steroidal antiinflammatory drug (NSAID) animal models [53]. Syringic acid has been shown to have hepatoprotective activity, which may contribute to the effective treatment of jaundice using ICM [54] because one of the pathogenesis of jaundice is closely related to the role of liver.

The main flavonoids identified in this study were kaempferol, quercetin, and apigenin, and their derivatives. Except apigenin, the other components were identified for the first time in ICM or even in the *Incarvillea* genus. Many reports indicate that kaempferol and apigenin exert their anti-inflammatory effects through different mechanisms and targets. For instance, kaempferol inhibits the expression of cyclooxygenase-2 (COX-2) by reducing sarcoma (SRC) kinase activity; quercetin can inhibit the gene expression of tumor necrosis factor-α (TNF-α) by regulating the level of NF-kB (nuclear factor kB) in peripheral blood mononuclear cells [55,56,57]. Those mechanisms will probably be the pathways through which ICM treats tympanitis in traditional medicine.

In this study, three alkaloids (incarvilline, plantagonine, and protopine) were identified. Protopine has not been identified in the *Incarvillea* genus before. Some articles mention that, in addition to its common antinociceptive effect like other alkaloids, protopine can inhibit inflammation [58]. This suggests that protopine may provide some of the anti-inflammatory effect of ICM. Belonging to iridoid terpenoids, 8-epideoxyloganic has been found in *Incarvillea arguta* and *Incarvillea delavayi* before. In these experimental studies, oral 8-epideoxyloganic acid showed weak anti-nociceptive activity [12,59]. Besides alkaloids, this component may also have analgesic effects in this plant. 

Organic acids found in this study, which are reported to exist in all plants, have never been studied in the *Incarvillea* genus, are beneficial to plant growth. Esculetin belongs to the coumarins, which have also not been found in this genus before. Esculetin has been reported to have antioxidant activity in vivo and to reduce inflammation [60,61]. 

These results suggest that the pharmacological activity of ICM may closely related to the synergistic action of these components because of the integrity of traditional Chinese medicine in exerting its pharmacodynamics.

### 3.3. Antioxidant Activity In Vitro

The above methods for determining antioxidant capacity are based on different mechanisms. Previous studies have suggested that DPPH, ABTS, and reducing power are based on electron transfer, whereas β-carotene bleaching tests are based on hydrogen atom transfer [62]. Summarizing the above results, we found that all the extracts have certain antioxidant activity and wICM showed better antioxidant activity in all tests, except for the DPPH assay. Previous studies showed that the contents of phenolic acids and flavonoids are positively correlated with antioxidant capacity [63]. However, the content of various components in mICM was the highest in the component quantification section of this paper. Therefore, other antioxidant components may exist in ICM besides alkaloids, flavonoids, and phenols. For example, the iridoid terpenoids and organic acids found in the qualitative identification section of this study have been proven to have definite antioxidant activity [64,65]. Other studies found that steroids, phenylethanoid glycosides, and sesquiterpenoids in the *Incarvillea* genus [7,66]. These classes of compounds from different plants in the same genus, have been reported to be closely related to antioxidant activity, and may also exist in ICM and play an antioxidant role [67,68,69]. 

### 3.4. Analgesic Effect

Analgesic models can be divided into two categories according to the modelling method: physical stimulation and chemical stimulation. According to the different mechanisms of action, there are peripheral analgesia models and central analgesia models [70]. Although analgesics can relieve different types of pain, their mechanisms of action vary. Therefore, using only a single animal model to evaluate the efficacy of an analgesic would be ineffective. Three in vivo pain models were used in this study. The first one was the formalin test with neuronal pain (first phase) and inflammatory pain (second phase). Because acetic acid induces pain by indirectly releasing endogenous mediators, such as prostaglandin E2 (PGE2), to stimulate nociceptive neurons, it is often used in the screening of peripheral analgesic drugs [71]. The hot plate method is a physical stimulus reaction involving the advanced central nervous system, which can reflect the central analgesic effect [72]. 

According to the experimental results, the mice given ICM extract were less harmed compared with the mice of the control group in different models. This suggests that ICM can be used as a potential source of analgesics, just like other plants of the *Incarvillea* genus [10]. In the formalin test, wICM had a stronger inhibitory effect on the second phase because the low concentration of wICM was already effective in the second period and only high concentration of wICM produced curative effects in the first period. Similarly, in the acetic acid writhing test, the lowest concentration of wICM also played an anti-nociceptive role. However, in the hot plate experiment, the extract showed a protective effect on mice in the medium concentration group. Therefore, wICM may be more effective for peripheral analgesia, and its mechanism may be related to the inhibition of inflammatory mediators release [73]. Previous studies of other plants of the same genus have reported that their analgesic activity is mainly due to the presence of alkaloids [10] and iridoid terpenoids also contribute to the anti-nociceptive activity of plants in the genus Incarvillea [12]. Therefore, combined with the previous research results and the components analysis results of this study, we speculat that the analgesic activity of ICM is mainly related to alkaloids and iridoid terpenoids. The specific material basis of analgesic activity needs to be verified by subsequent work.

## 4. Materials and Methods

### 4.1. Chemicals Reagents and Plant Materials

Approximately 2 kg samples of the aerial parts of ICM were freshly collected from Banma County (Qinghai, China) in August 2015 and were identified and authenticated by Professor Lijuan Mei (Northwest Institute of Plateau Biology, Chinese Academy of Science, Xining, China). The plant materials were shade-dried, ground into powder, sieved through a 0.8-mm metal sieve to achieve a standard size of particles and stored at 4 °C until use. DPPH, ABTS, BHT, VC, β-carotene, Tween 40, and linoleic acid were obtained from Aladdin Co. (Los Angeles, CA, USA). Ferric chloride (FeCl_3_·6H_2_O), potassium ferricyanide [K_3_Fe(CN)_6_] and other chemicals used were purchased from Sinopharm Chemical Reagent (Shanghai, China). The standards of gallic acid, rutin, and berberine were purchased from the National Institutes for Food and Drug Control (Beijing, China).

### 4.2. Sample Preparation and Extractions

ICM powder samples (5 g) were extracted with methanol, water, and acetone (50 mL). An ultrasonic bath at a frequency of 70 kHz was used to extract ICM for 30 min. The extraction procedure was conducted twice more, and the extracts were filtered. The filtrates were combined, and the solvents were evaporated using rotary evaporation under reduced pressure. The residues obtained were dissolved and diluted to 25 mL with the corresponding solvent. The concentrations of mICM, wICM, and aICM were all 0.2 g/mL (*w*/*v*). Finally, the samples were stored at 4 °C to analyze the TAs, TFs, and TPs contents and the in vitro antioxidant activities.

### 4.3. Determination of The Total Alkaloids Content

The acid dye colorimetric method was used to determine the TAs content of the sample extracts [74]. First, 0.5 g of bromocresol green was dissolved in a NaOH solution (50 mL; 0.2 M). Sodium acetate (9 g) was dissolved in 11.5 mL glacial acetic acid and diluted with ultrapure water to 500 mL. Then, bromocresol green solution (25 mL) was added to a volumetric flask and diluted with 500 mL sodium acetate buffer. After its pH value was adjusted to 4.12, the bromocresol green buffer was prepared. The sample solutions of various concentrations (1 mL) were removed from their solvents and dissolved in the bromocresol green buffer (10 mL). Afterward, the mixture was transferred to a separatory funnel with chloroform (4 mL) and shaken vigorously for 2 min. The extraction was conducted another 2 times. The layers of chloroform were mixed together, and the absorbance was measured against the blank at 435 nm. The TAs content was calculated on the basis of the calibration curve of berberine, and the results are expressed as mg of berberine per gram of dry weight.

### 4.4. Estimation of The Total Flavonoids Content

The TFs content of ICM was detected using the AlCl_3_ spectrophotometric method with modifications [75]. Briefly, 2 mL sample solutions and 50% ethanol (8 mL) were transferred into a 25-mL volumetric flask, and 1 mL 5% NaNO_2_ solution was added and shaken vigorously. After 6 min, 1 mL 10% Al(NO_3_)_3_ was evenly mixed and incubated for 6 min. Four percent NaOH (10 mL) was added, and the mixture was diluted to the volume with ultrapure water. The absorbance at 510 nm was detected after 15 min using a spectrophotometer (Shimadzu UV-1750, Kyoto, Japan). Rutin was used to plot the standard curve, and the results are expressed as mg of rutin equivalent per gram of extract.

### 4.5. Measurement of the Total Phenols Content

The TPs content was measured using a slightly modified colorimetric method [76]. Each sample (0.5 mL) was added to a 25 mL brown volumetric flask with 7 mL 50% ethanol, followed by the addition of 2 mL of a 0.3% sodium dodecyl sulfate solution and 2 mL of a mixed solution of 0.5% potassium ferricyanide-1% ferric chloride (1:1, *v*/*v*). After 5 min of incubation in the dark, the mixture was diluted with 0.1 M hydrochloric acid solution and incubated for an additional 20 min in the dark. The absorbance was measured at 760 nm against an ultrapure water blank on a spectrophotometer. Gallic acid was used as a standard, and TPs content is expressed in milligrams equivalents of gallic acid per gram of each fraction.

### 4.6. LC-ESI-QTOF-MS/MS Analysis

The methanol extracts of the samples were analysed by LC-ESI-QTOF-MS/MS (AB Sciex, Framingham, MA, USA) in both the positive and negative ion modes. The optimum ESI operational conditions are as follows: capillary voltage, 5.5 kV (ESI^+^) or −5.5 kV (ESI^−^); source temperature, 600 °C; nebulizer gas, N_2_, 55 psi; and scan range, 50 to 1000 *m*/*z*. The injection volume was 3 μL and the total flow rate was 0.2 mL/min. A gradient mobile phase of water (A) and acetonitrile (B) was employed for 0–20 min, 15–20% B; 20–45 min, 20–40% B; 45–65 min, 40–60% B; 65–75 min, 60–80% B; and 75–80 min, 80–15% B.

### 4.7. In Vitro Antioxidant Activities

#### 4.7.1. Scavenging Effect on DPPH Radicals

DPPH radical-scavenging activities of the three extracts were determined using the method followed by Jeong et al. [77]. The methanolic solution (0.8 mL; 0.09 mM) of DPPH was added to the solutions (0.2 mL), prepared with various extracts of different concentrations and stirred. Afterward, the mixture was incubated for 60 min in the dark, and the absorbance was determined at 517 nm. Ultrapure water was used as the blank control instead of the extract, and VC and BHT were used as the positive controls. The scavenging activity of the DPPH free radicals was calculated as follows:

Scavenging effect (%) = (1 − A_DPPH-sample_/A_DPPH-control_) × 100%

where A_DPPH-control_ is the absorbance of the control (DPPH solution without sample) and A_DPPH-sample_ is the absorbance of the test sample. The IC_50_ was calculated using the linear relation between the compound concentration and the probability of the scavenging effect of DPPH radicals.

#### 4.7.2. Scavenging Effect on ABTS Radicals

Free radical scavenging activities of the plant extracts at different concentrations were determined following the method of Re et al. [78]. The ABTS radical cation (ABTS^+^) was formed by the filtering mixture, which was produced by reaction of ABTS stock solution and MnO_2_, through a 0.2 μm polyvinylidene fluoride (PVDF) membrane. To adjust the absorbance of ABTS^+^ solution at 734 nm to 0.70 ± 0.02, 0.01 M phosphate-buffered saline (PBS, pH 7.4) solution was used for dilution. We evenly mixed the samples with different concentrations (0.05 mL) and ABTS^+^ solution (3 mL), and kept them away from light for 6 minutes at room temperature. Finally, the absorbance of the mixture was determined at 734 nm against the control, which contained ultrapure water instead of the test sample. VC and BHT are used as the positive controls. The results were expressed according to the following formula:

Scavenging effect (%) = (1 − A_ABTS-sample_/A_ABTS-control_) × 100%

where A_ABTS-control_ is the absorbance of control and A_ABTS-sample_ is the absorbance of samples. The IC_50_ values of the extracts, i.e., the concentration of extract necessary to decrease the initial concentration of ABTS^+^ by 50%, were also calculated. 

#### 4.7.3. Reducing Power Test

The reducing power was conducted using the method described by Wang et al. with some modifications [79]. One milliliter samples at various concentrations were mixed with phosphate buffer (2 mL; 0.2 M; pH 6.8) and potassium ferricyanide (2 mL; 1%). After the mixture was incubated at 50 °C for 20 min and immediately cooled, trichloroacetic acid (2 mL) was added, mixed evenly and centrifuged at 3000 rpm for 10 min. We removed the upper layer (2 mL) and mixed it with ultrapure water (2 mL) and ferric chloride (1 mL; 0.1%). After 10 min, the absorbance at 700 nm was read, where a higher absorbance indicated a stronger antioxidant activity. VC and BHT were used as the positive controls. 

#### 4.7.4. β-Carotene Bleaching Test

Antioxidant activity was determined by measuring the inhibition of volatile organic compounds and the conjugated diene hydroperoxides resulting from linoleic acid oxidation [80]. We mixed 500 μL of β-carotene (1 mg/mL dissolved in chloroform) with linoleic acid (0.2 mL; 0.1 g/mL dissolved in chloroform) and Tween 40 (1 mL; 0.2 g/mL dissolved in chloroform). Chloroform was evaporated at 50 °C. Afterward, ultrapure water (100 mL) was added with vigorous shaking. The reaction mixture (5 mL) was mixed with various concentrations (0.1 mL) of the extracts to form a fresh mixture, which was incubated for 2 h in a 50 °C water bath in the dark. The BHT group (the positive control) and the control group replaced the test sample with water and performed the same operation. The absorbance at 470 nm was measured every 25 min. Antioxidant activity (AA) was calculated using the following equation:

AA(%) = (A_s(120)_ − A_c(120)_/(A_c(0)_-A_c(120)_) × 100

where A_s(120)_ and A_c(120)_ are the absorbance of the sample and the control, respectively, at *t* = 120 min, and A_c(0)_ is the absorbance of the control, at *t* = 0 min.

### 4.8. Biochemical Assay

#### 4.8.1. Animals

A total of 65 Kunmin mice of SPF grade weighing 18–20 g were used in the experimental studies, purchased from the animal experiment center of Chongqing Medical University. The mice were maintained at constant room temperature (22 ± 2 °C) under a 12 h light/dark cycle with free access to standard food and water. Animal experiments were conducted following the international guidelines, and the experimental procedures were approved by the Animal Ethics Committee, Chongqing Medical University, China (NO. 2017017). 

#### 4.8.2. Formalin Test

The formalin test was conducted as described by Chio et al. [81]. Groups, containing male and female mice (a total of 5 in each group), were treated orally with distilled water (control group) and different concentrations (15, 30, and 60 g/kg) of wICM (low-, mid- and high-concentration groups) for 7 days. Sixty minutes after the last treatment, formalin (10 µL, 2.5%) was subcutaneously injected into the left hind paw, and the behavior of mice was observed. The nociceptive score was measured in the first phase (0–10 min) and the second phase (20–30 min), where 0 = walking normally; 1 = the injected paw placed gently on the floor and claudication; 2 = lifting of the injected paw; and 3 = licking, biting or shaking of the processed paw [82,83].

#### 4.8.3. Hot-Plate Test

To determine central analgesic activity, the hot-plate test, according to the procedure described by Jarogniew et al. was used with minor modifications [83]. Female mice were selected for the experiment. The mice were placed on a hot plate at 55 ± 0.5 °C, and the response times of licking the hind paw were recorded. The mice with pre-drug latencies between 10 and 30 s were chosen for further study. Animals (five per group) were pre-treated with distilled water, diclofenac sodium (0.03 mg/g), and different doses of wICM (15, 30, or 60 g/kg) for a week. Thirty minutes after the last treatment, they were placed on the hot plate, and the latency (the time when the mice began licking the hind paw) was measured. The cut-off time was set to 30s to minimize hind paw damage.

#### 4.8.4. Acetic Acid-induced Writhing Test

The male mice were divided into four groups of 5 animals each. The control mice received distilled water by intragastric administration (10 mL/kg), and the test mice received different concentrations of wICM (15, 30, or 60 g/kg) for 7 days. Sixty minutes after the last treatment, 2 mL of 0.7% acetic acid solution were injected i.p. The number of writhing reactions, including abdominal contractions, hind limb extensions, body twists, and hip elevations, was counted for 10 min [84]. The analgesic rate was calculated using the following equation:

Analgesic rate (%) = [1 − N_ICM_/N_control_] × 100%

where N_ICM_ and N_control_ are the numbers of writhing behaviors in the groups treated with wICM and water, respectively.

### 4.9. Statistical Analysis

All experiments were performed in triplicate, and all data are expressed as the mean ± standard deviation (SD). One-way analysis of variance (ANOVA) was used for the statistical analysis using SPSS 17.0 (SPSS Inc., Chicago, IL, USA). Values of *p* < 0.05 were considered to be statistically significant. Photoshop CS6 (Adobe Systems Inc., San Jose, CA, USA) and origin 7.5 (OriginLab, Northampton, MA, USA) were used to create the figures.

## 5. Conclusions

Chemical analysis was used to identify a total of 25 compounds, including phenolic acids, flavonoids, alkaloids, and others. Compounds **1**–**23** were reported for the first time from ICM and except for compounds **19** to **23** and **14**, they were found for the first time in the *Incarvillea* genus as well. The TPs, TFs, and TAs contents in different extracts were calculated, which revealed that mICM contains the highest concentrations of these compounds. In the antioxidant experiments, although all the extracts showed certain abilities to scavenge or reduce free radicals, making ICM a potential source of antioxidants, water extracts showed the best antioxidant activity in all tests except for DPPH assay. It’s indicated that methanol can be used as extraction solvent if only more phenols or alkaloids are wanted, but water can be used as solvent if samples with strong biological activity are wanted. The analgesic tests indicated that the constituents of wICM minimize sustained pain and possess more peripheral analgesic activity. Combined with the results of the component analysis and previous study, we speculate that the analgesic activity of ICM may be mainly attributed to alkaloids and iridoid terpenoids. This biological and chemical information can provide a reference for subsequent study and can be useful for the exploitation and use of ICM in the future.

## Figures and Tables

**Table 1:** TPs, TFs, TAs content of ICM extracted with different solvents.

Solvents	Total Phenols (GAE mg/g DW)	Total Flavonoids (RE mg/g DW)	Total Alkaloids (BE mg/g DW)
Water	22.00 ± 0.80^c^	29.24 ± 0.09^c^	1.05 ± 0.02^c^
Methanol	29.24 ± 0.04^b^	49.07 ± 0.25^b^	5.01 ± 0.07^b^
Acetone	11.28 ± 0.05^a^	9.11 ± 0.08^a^	2.43 ± 0.02^a^

Values are expressed as mean ± standard deviation; different superscript lowercase letters denote statistically significant difference (*p* < 0.05).

**Table 2:** Identification of the compounds in the mICM by LC-ESI-QTOF-MS-MS.

Peak No.	Rt (min)	Molecular Formula	[M + H]^+^ (*m*/*z*)	[M − H]^−^ (*m*/*z*)	Error (ppm)	MS/MS Fragments	Proposed Compound	Reference	Classification
1	6.88	C9H10O5		197.04555	−2.1	182.024,167.0005	Syringic acid	[18]	Phenolic acids
2	5.58	C7H6O3		137.02442	0.4	108.0002, 92.0266	*p*-Hydroxybenzoic acid	[19]	Phenolic acids
3	2.02	C7H12O6		191.05611	−1.1	173.0461,127.0410 93.0350, 85.0300, 59.0145	Quinic acid	[20]	Phenolic acids
4	2.13	C13H16O9		315.0721	0.7	153.0568, 59.0159	Protocatechuic acid hexoside	[21]	Phenolic acids
5	12.54	C27H30O16		609.14611	−0.5	301.0347 (47.96)	Rutin	[22]	Flavonoid
6	18.12	C27H30O15		593.15119	−0.1	285.0395	Kaempferol-3-*O*-rutinoside (nicotiflorin)	[23]	Flavonoid
7	14.7	C21H20O12		463.0882	−2	301.0345	Quercetin-3-*O*-glucoside	[24]	Flavonoid
8	20.43	C21H20O11		447.09329	−2.4	285.0384	Kaempferol-3-*O*-glucoside (astragalin)	[24]	Flavonoid
9	21.87	C21H20O10		431.09837	−5	269.0424	Apigenin-7-*O*-glucoside (apigetrin)	[25]	Flavonoid
10	2	C27H30O16		609.1461	−0.5	301.0347	Quercetin-3-glucoside-7-*O*-rhamnoside	[26]	Flavonoid
11	8.31	C20H19NO5	354.1336		−1	206.0804,188.0694 149.0596	Protopine	[27]	Alkaloid
12	12.54	C27H30O16		609.1458	−0.5	301.0347,271.0235	Quercetin-3-*O*-robinobioside	[28]	Flavonoid
13	19.34	C16H12O7	317.9656		−0.4	317.0645,302.0421 285.0360,274.0463	Isorhamnetin	[29]	Flavonoid
14	12.59	C15H10O7	303.0497		−0.7	303.0484,285.0411 229.0498,153.0188	Quercetin	[29]	Flavonoid
15	14.73	C21H20O12	465.1028		−1.2	303.0485	Quercetin-3-glucoside	[29]	Flavonoid
16	2.37	C4H6O5		133.01425	1.2	115.0038,71.0155, 51.0191	Malic Acid	[23]	Organic acid
17	2.61	C6H8O7	303.04993	191.01973	−2	111.0088,87.0089, 59.0154	Citric acid	[22]	Organic acid
18	6.36	C9H6O4		177.01933	−0.7	149.0281, 133.0274, 105.0337, 89.0396	Esculetin	[18]	Coumarins
19	1.69	C11H21NO	184.16959		0.6	184.1691,166.1585,107.0851	Incarvilline	[30]	Alkaloid
20	2.56	C10H11NO2	178.0863		0.7	162.0543,132.0797,118.0643,117.0568	Plantagonine	[31]	Alkaloid
21	10.17	C16H24O9	359.1348		−0.7	197.0810,153.0922	8-Epideoxyloganic acid	[32]	Iridoids
22	11.5	C9H8O3		163.04007	0.3	135.0455, 89.0397	Coumaric acid	[33]	Phenolic acids
23	3.6	C7H6O4		153.01933	0.3	109.0295,81.0366, 53.0414	Protocatechuic acid	[34]	Phenolic acids
24	6.59	C9H8O4		179.03498	0.9	136.0514	Caffeic acid	[33]	Phenolic acids
25	38.26	C15H10O5		269.04555	−3.4	117.0351, 151.0046	Apigenin	[35]	Flavonoid

**Table 3:** The analgesic rate of wICM in the acetic acid test.

Groups	Analgesic Rate (%)
Low-dose	25.05%
Mid-dose	32.79%
High-dose	58.20%
